# In Vivo and In Vitro Evaluation of Urinary Biomarkers in Ischemia/Reperfusion-Induced Kidney Injury

**DOI:** 10.3390/ijms222111448

**Published:** 2021-10-23

**Authors:** Keiko Hosohata, Denan Jin, Shinji Takai

**Affiliations:** 1 Education and Research Center for Clinical Pharmacy, Osaka Medical and Pharmaceutical University, Osaka 569-1094, Japan; 2 Department of Innovative Medicine, Osaka Medical and Pharmaceutical University, Osaka 590-0906, Japan; denan.jin@ompu.ac.jp (D.J.); shinji.takai@ompu.ac.jp (S.T.)

**Keywords:** sensitive biomarker, renal tubular damage, ischemia-reperfusion

## Abstract

Oxidative stress plays an important role in the pathophysiology of acute kidney injury (AKI). Previously, we reported that vanin-1, which is involved in oxidative stress, is associated with renal tubular injury. This study was aimed to determine whether urinary vanin-1 is a biomarker for the early diagnosis of AKI in two experimental models: in vivo and in vitro. In a rat model of AKI, ischemic AKI was induced in uninephrectomized rats by clamping the left renal artery for 45 min and then reperfusing the kidney. On Day 1 after renal ischemia/reperfusion (I/R), serum creatinine (SCr) in I/R rats was higher than in sham-operated rats, but this did not reach significance. Urinary N-acetyl-β-D-glucosaminidase (NAG) exhibited a significant increase but decreased on Day 2 in I/R rats. In contrast, urinary vanin-1 significantly increased on Day 1 and remained at a significant high level on Day 2 in I/R rats. Renal vanin-1 protein decreased on Days 1 and 3. In line with these findings, immunofluorescence staining demonstrated that vanin-1 was attenuated in the renal proximal tubules of I/R rats. Our in vitro results confirmed that the supernatant from HK-2 cells under hypoxia/reoxygenation included significantly higher levels of vanin-1 as well as KIM-1 and NGAL. In conclusion, our results suggest that urinary vanin-1 might be a potential novel biomarker of AKI induced by I/R.

## 1. Introduction

Acute kidney injury (AKI) is commonly encountered in clinical settings. It is associated with morbidity and mortality in hospitalized patients [1]. The most common causes of AKI are drugs [2], contrast medium [3], sepsis [4], heart failure [5], chronic liver disease [6], hypoperfusion/shock [7], and ischemia [8]. In particular, ischemia is an important underlying pathophysiological mechanism in many forms of AKI [9]. Ischemic insult is increased when there is reduced blood flow to the kidney after drug or toxicant exposure or as a component of vascular disease, sepsis, or volume depletion and hypotension [10]. Following the recovery of blood flow after ischemia, productions of reactive oxygen species (ROS) and reactive nitrogen species within mitochondria are markedly enhanced.

ROS play a major role in the promotion of inflammatory responses in the progression of renal ischemia/reperfusion (I/R)-injury. Focusing on vanin-1, which is a tissue sensor of oxidative stress, we reported that vanin-1 was shed into the urine prior to increases of conventional biomarkers such as N-acetyl-β-d-glucosaminidase (NAG), serum creatinine (SCr), or blood urea nitrogen (BUN) in rats with nephrotoxicant- and drug-induced renal tubular injury [11,12]. In addition, similar findings were observed in cancer patients treated with cisplatin [13]. Vanin-1 is a glycosylphosphatidylinositol (GPI)-anchored protein in cell membranes [14,15]. It is also an ectoenzyme that hydrolyzes pantetheine to pantothenic acid (vitamin B5) and cysteamine. In turn, cysteamine, which is a low-molecular thiol, is then converted to cystamine which inhibits γ-glutamylcysteine synthetase (γGCS), the rate-limiting enzyme of glutathione synthesis [16]. Furthermore, vanin-1 knockout mice exhibited resistance to oxidative stress, as well as down-regulated tissue inflammation, thereby leading to lower levels of oxidative tissue damage. As a result, the survival of vanin-1 knockout mice has been improved [17].

Recently, kidney injury molecule-1 (KIM-1) and neutrophil gelatinase-associated lipocalin (NGAL) were developed as renal biomarkers promoting the early diagnosis of AKI. KIM-1, a type I transmembrane glycoprotein, is rapidly upregulated on the apical surface of proximal tubules at gene and protein levels after AKI. NGAL, which is involved in metabolic homeostasis, apoptosis, infection, the immune response, and inflammation, is detected at the onset and progression of inflammatory diseases including AKI [18,19].

Afterwards, we examined whether vanin-1 is involved in renal tubular injury using a renal I/R-induced AKI model and compared it with profiles of urinary biomarkers such as KIM-1 and NGAL.

## 2. Results

### 2.1. Renal Pathological Change

**Figure 1:**
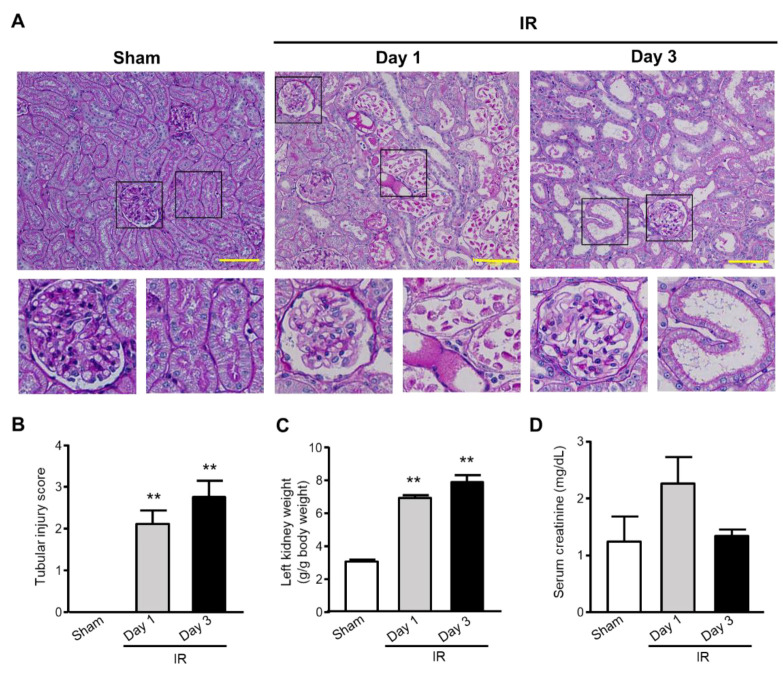
Kidney injury was determined by ischemia reperfusion in rats with unilateral nephrectomy. (**A**) Representative photomicrographs of the renal cortical regions stained by periodic acid–Schiff (PAS) reagent. Lower panels are enlarged images of upper panels (left, glomeruli; right, tubules). (**B**) Renal tubular injury (dilatation) was assessed using a semiquantitative score from 0 to 4: 0 = normal; 1 = mild; 2 = moderate; 3 = severe; and 4 = very severe. (**C**,**D**) Left kidney weight (**C**) and serum creatinine (**D**) were assessed. Sham-operated rats, *n* = 6; I/R-treated rats, *n* = 5. ** *p* < 0.01 vs. sham-operated rats. Scale bar, 100 μm.

Animal experiments using male Sprague–Dawley rats consisted of sham-operated controls, I/R-rats that were sacrificed on Day 1 for histologic examination, and I/R-rats that were sacrificed on Day 3. In this study, ischemia was induced by clamping the left renal artery in rats which underwent right nephrectomy 14 days prior to ischemia, and reperfusion was induced 45 min later. Representative photomicrographs of renal cortical sections stained with periodic acid–Schiff (PAS) reagent are shown in Figure 1A. Sham-operated rats remained intact or had very slight renal tubular damage, if any. On the other hand, I/R-treated rats showed renal injuries, including loss of the brush border, vacuolization in tubular cells, and cast formation (Figure 1A), which were confirmed by scoring of the histological sections in terms of degeneration (Figure 1B). On Days 1 and 3 after I/R treatment, the kidney weight-to-body ratios of I/R-treated rats were significantly higher than those of sham-operated rats (Figure 1C). On the other hand, renal I/R-treated rats showed an increase in SCr on Day 1, although this did not reach significance, and its increase declined to the same level as that of sham-operated rats (Figure 1D).

### 2.2. Evaluation of Renal Biomarkers and Urinary Vanin-1

**Figure 2:**
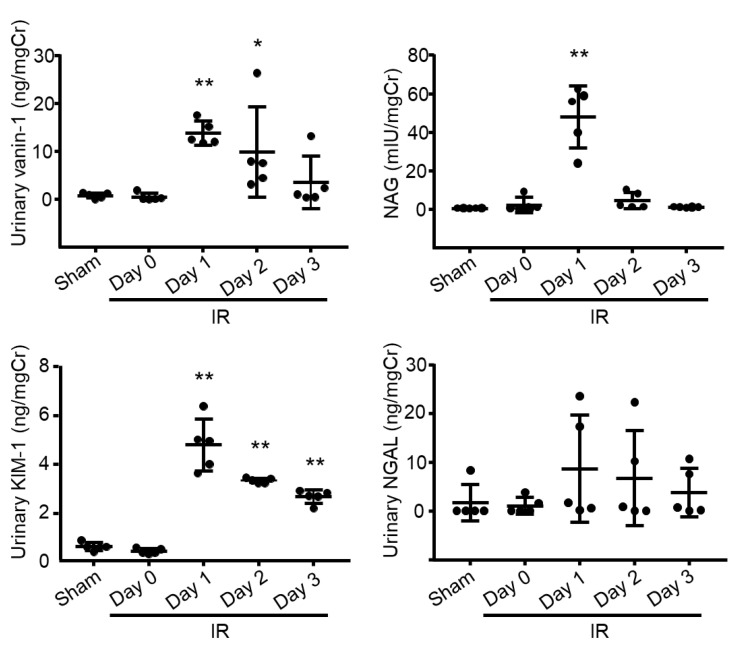
Evaluation of renal biomarkers. Concentrations of urinary vanin-1, NAG, KIM-1, and NGAL were normalized to the urinary creatinine concentration. Sham-operated rats, *n* = 6; I/R-treated rats, *n* = 5. * *p* < 0.05; ** *p* < 0.01 vs. sham-operated rats.

As shown in Figure 2, urinary vanin-1 was significantly elevated on Day 1 and maintained at a significantly high level on Day 2 after I/R-treatment; however, the values showed no significance on Day 3. After renal I/R-treatment, NAG showed a peak level on Day 1; however, on Days 2 and 3, it was reduced to almost the same levels as on Day 0 and in sham-operated rats. Urinary KIM-1 showed a peak level on Day 1 and decreased on Days 2 and 3 with significance. Urinary NGAL showed wide variation in individual animals on Days 1 and 2, attributed to there being no significant difference among groups.

### 2.3. Localization and Expression of Vanin-1 in the Kidney

**Figure 3:**
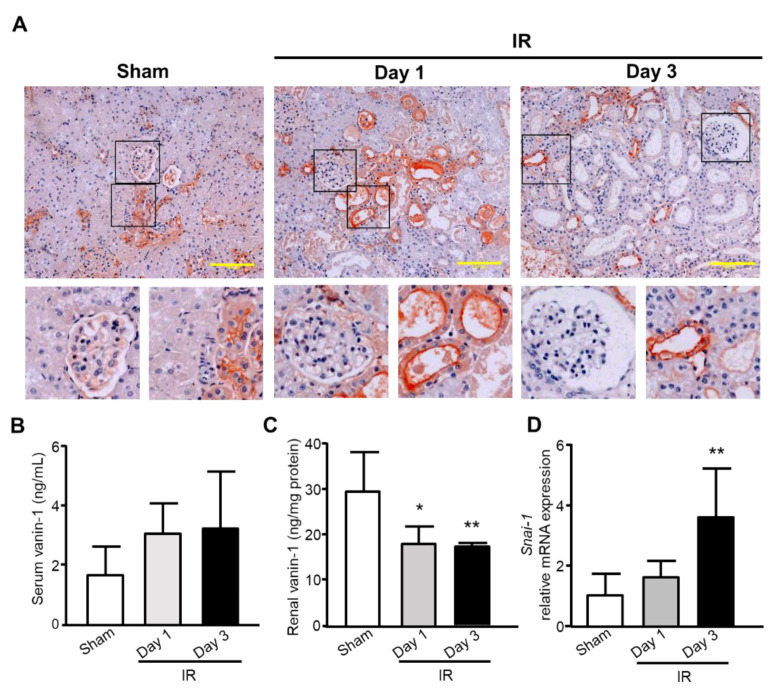
Evaluation of renal and serum vanin-1 and progression to EMT after I/R-treatment. (**A**) Representative immunostaining of vanin-1 in the kidney of rats. Vanin-1 expression was localized both around the injured tubules and within a subset of injured tubules, but not in the glomeruli. Scale bar, 100 μm. (**B**,**C**) Serum and renal vanin-1 were determined. (**D**) The mRNA level of snail family zinc finger 1 (*Snai1*) was determined using real-time quantitative PCR. The mRNA expression level was normalized to that of *Gapdh*. Sham-operated rats, *n* = 6; I/R-treated rats, *n* = 5. * *p* < 0.05; ** *p* < 0.01 vs. sham-operated rats.

As shown in Figure 3A, immunofluorescence analysis showed that vanin-1 was localized in proximal tubules, but not glomeruli. In addition, immunofluorescence intensity of vanin-1 was strong on the apical side, suggesting that vanin-1 originates from the apical side of renal proximal tubules and is shed into urine. In addition, vanin-1 protein was weakly but diffusely expressed in the intact proximal tubules of renal cortices of normal rats (Figure 3A). Of note, the intensity of vanin-1 was clearly strong only in the injured tubules on Day 1 after I/R-treatment, which was sparsely observed only in severely damaged tubules on Day 3. As for the serum concentration of vanin-1, it was slightly increased in the groups with I/R-treatment, but the differences did not reach significance (Figure 3B). On the other hand, renal protein expression of vanin-1 significantly decreased on Days 1 and 3 (Figure 3C). Therefore, it is suggested that urinary vanin-1 was leaked from renal tissues at least in the AKI models we examined. Next, we examined the mRNA expression of snail family zinc finger 1 (*Snai1*), which is involved in epithelial-mesenchymal transition (EMT) (Figure 3D). Interestingly, *Snai1* mRNA expression significantly elevated over time during I/R-treatment, suggesting that EMT should progress and production of renal vanin-1 in epidermal cells might decrease.

### 2.4. Hypoxia/Reoxygenation Release Vanin-1 in the Supernatant

**Figure 4:**
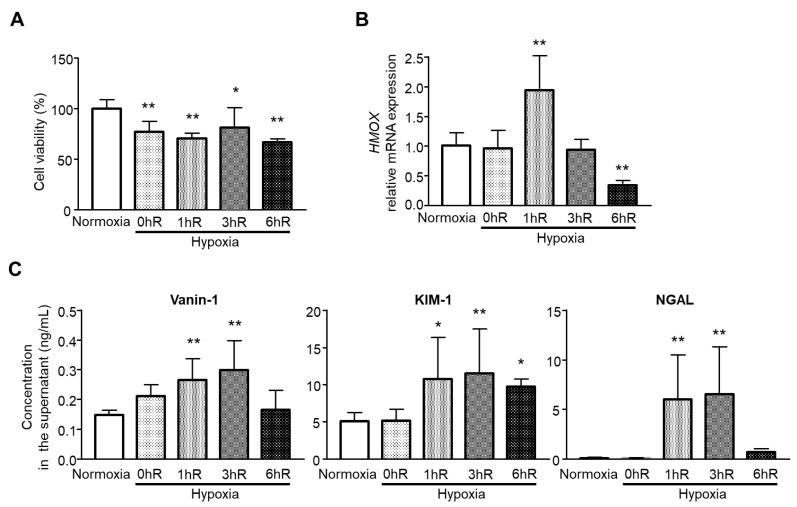
Effects of hypoxia reoxygenation on vanin-1, KIM-1, and NGAL in vitro. HK-2 cells were exposed to normoxia (control) or hypoxia for 24 h followed by reoxygenation for 0 h (0hR), 1 h (1hR), 3 h (3hR), or 6 h (6hR). (**A**) CCK8 assays were used to detect the cell viability of HK-2 cells under various conditions. (**B**) The mRNA level of *heme oxygenase 1* (*HMOX1*) was determined using real-time quantitative PCR. The mRNA expression level was normalized to that of *GAPDH*. (**C**) The supernatant from the cells (*n* = 6 per group) was collected for measurement of vanin-1, KIM-1, or NGAL. HK-2, human immortalized tubular epithelial cells. * *p* < 0.05; ** *p* < 0.01 vs. normoxia.

To examine whether the findings of in vivo experiments apply in vitro, human renal proximal tubular epithelial HK-2 cells were exposed to hypoxia followed by reoxygenation for different times of 0, 1, 3, or 6 h. As shown in Figure 4A, after hypoxia for 24 h, cell viability decreased significantly. Next, we examined whether different reoxygenation times affected oxidative stress levels. As shown in Figure 4B, heme oxygenase 1 (*HMOX1*) showed the highest mRNA expression at 1 h. Together, vanin-1 as well as KIM-1 and NGAL showed significantly higher levels in supernatant from HK-2 cells (Figure 4C).

## 3. Discussion

In the present study, we detected vanin-1 in the urine in the early stage of AKI development; on the other hand, SCr, a conventional biomarker, was elevated in the early stage of AKI but this did not reach significance. The conventional renal marker urinary NAG transiently elevated but rapidly dropped to a low level the same as that of sham despite histologically apparent renal injury. Urinary NGAL showed marked variability, whereas urinary KIM-1 maintained significance after the peak level. Our study clearly demonstrated that the elevation of urinary vanin-1 preceded that of SCr in the I/R-induced AKI model. Moreover, urinary vanin-1 was as sensitive as urinary KIM-1 in I/R-induced renal injury in rats, and vanin-1 may be a novel biomarker for the earlier detection of I/R-induced AKI, at least in an experimental model.

Conventional markers of renal injury such as SCr and BUN are not sensitive enough, especially in the initiation phase of AKI [20]. New biomarkers with accurate diagnostic values are therefore needed. In this study, urinary vanin-1 was characterized as a biomarker of the initiation phase of AKI. This finding is consistent with our previous report in which vanin-1 was a sensitive marker of drug-induced AKI [12]. Interestingly, we found that other biomarkers, such as NAG, NGAL, and KIM-1, showed differential patterns of urinary excretions: NAG showed a peak level on Day 1; however, on Days 2 and 3, it was reduced to almost the same levels as on Day 0 and in sham-operated rats. NGAL showed wide variation in individual animals on Days 1 and 2, attributed to there being no significant difference among groups. KIM-1 showed a peak level on Day 1 and decreased on Days 2 and 3 with significance. NGAL expression is induced in renal tubular cells during the regenerative process after kidney injury [21]; however, it is also expressed in other epithelial cells, neutrophils, and macrophages. Furthermore, plasma NGAL is also upregulated under several clinical conditions [21]. These could be explained by the wide variation in our study. Our in vitro results revealed that 24 h hypoxia (0hR) and reoxygenation for 1, 3, and 6 h decreased cell viability. Of note, *HMOX1*, which plays a key role in conferring cytoprotection against injury to renal epithelia, upregulated under oxygenation for 1 h after hypoxia followed by a decrease with reoxygenation over time, especially at 6 h, but not hypoxia (0hR). In line with the upregulation of *HMOX1*, vanin-1 as well as KIM-1 and NGAL in the supernatant from HK-2 cells significantly increased, and this persisted until 3 h for vanin-1 and NGAL. Similar to the in vivo results, NGAL showed wide variation in vitro.

Previously, we demonstrated a time-dependent decrease in renal cortical protein expression following drug-induced AKI in rodent models [12]. In this study, we found that renal vanin-1 protein time-dependently decreased 24 h after reperfusion in rats. This is partly because vanin-1 is expressed even in normal tissues in the kidneys of humans [22] and rodents [15]. We considered that urinary vanin-1 was leaked from renal tissues, at least in the I/R treatment AKI models we examined. In our results, *Snai1* mRNA expression significantly increased, which suggests that EMT progresses and the production of renal vanin-1 in epidermal cells might decrease. In the present study, the increase in urinary vanin-1 on Day 1 after I/R-treatment was significant, whereas that on Day 2 was slightly but significantly decreased, and it was not significant on Day 3, suggesting that renal vanin-1 was starting to leak into urine on Day 1 and continued to leak at least until Day 3. As a result, the decrease in the vanin-1 protein level might become clear on Day 3. Thus, urinary vanin-1 is a useful biomarker for the initiation phase of AKI, at least when examined using our I/R model, but it is not suitable for the maintenance phase of AKI.

Considering the feature of vanin-1 that its renal protein was decreased with the progression of proximal tubular damage in AKI due to the decrease in surviving proximal tubules, it is difficult to obtain the beneficial effects of vanin-1 inhibition for the prevention of kidney injury following I/R-injury because of the decrease in vanin-1 protein. Indeed, a recent study showed that pharmacological inhibition of vanin-1 is not protective in models of acute and chronic kidney injury using ischemia-reperfusion injury mice [23].

Previous reports on the association of oxidative stress with renal injury mostly involved glomerular injury. However, oxidative stress is also associated with tubular injury. Importantly, tubular injury also affects the glomerular filtration function via tubuloglomerular feedback [24]. In addition, renal tubules play a pivotal role in the maintenance of body fluid homeostasis and biological defense against toxic actions via absorption and secretion of various xenobiotics and endogenous compounds. Thus, it is important to detect renal injury at an early stage, start intervention, and follow the ameliorating effect using renal tubular biomarkers.

These studies provide evidence that urinary vanin-1 increases in rats subjected to I/R-induced AKI and that this increase correlates with renal pathological injury. We found that vanin-1 decreased in response to renal ischemia, which might contribute to the elevated urinary excretion of vanin-1 in rats with IR injury. The present data suggest that urinary vanin-1, which is leaked from tubular cells, is an earlier and more sensitive biomarker of I/R-induced AKI compared with SCr. Considering the distinct features of vanin-1 compared with those of other biomarkers, the combined use of vanin-1 with KIM-1 or NGAL will be more useful for the diagnosis of AKI and prediction of damaged renal sites.

## 4. Materials and Methods

### 4.1. In Vivo Experiments

#### 4.1.1. Animals and Experimental Protocol

**Figure 5:**
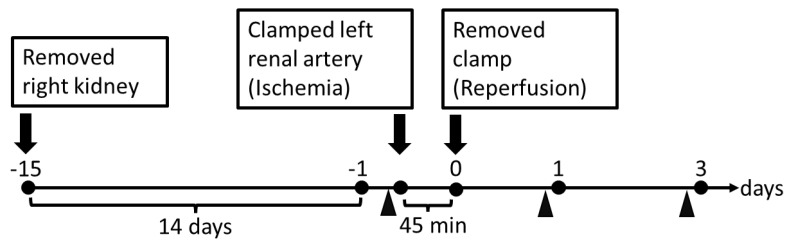
Experimental protocol for IR-induced AKI. Rats were placed in metabolic cages for a 12 h period in order to collect urine samples every day. All urine samples were stored at −80 °C until use. On Days 1 and 3, the rats were anesthetized with isoflurane to obtain blood and kidney tissues, respectively. Arrowheads indicate urine sampling time points.

All procedures complied with the ARRIVE guidelines and conformed to the Guide for the Care and Use of Laboratory Animals published by the US National Institutes of Health. The Experimental Animal Research Committee of Osaka Medical and Pharmaceutical University provided ethical approval for the laboratory animals used in this study (Permit No: 2020-022). Male Sprague–Dawley rats (200–250 g, Japan SLC, Shizuoka, Japan) were housed under a 12 h dark/light cycle and given regular food (CE-2; CLEA Japan, Tokyo, Japan) and water *ad libitum*. Two weeks before the study commenced, the right kidney was removed from rats through a small flank incision under isoflurane anesthesia (2~3%). After a 2-week recovery period, uninephrectomized rats were used to induce ischemic AKI: the left kidney of pentobarbital (50 mg/kg, i.p.)-anesthetized rats was exposed through a small flank incision and the left renal artery was occluded with a non-traumatic clamp for 45 min. At the end of the ischemic period, the clamp was released to allow reperfusion (Figure 5).

#### 4.1.2. Histological Analysis

After kidney tissues were fixed with Carnoy Solution (Muto Pure Chemicals Co., Ltd., Tokyo, Japan) for 24 h and embedded in paraffin, the sections (4 μm) were stained with periodic acid–Schiff (PAS) as previously detailed [25]. Tubular dilatation was graded as 0–4 (grade 0, normal; grade 1, mild; grade 2, moderate; grade 3, severe; and grade 4, very severe) using semiquantitative scoring [26]. An average score of 10 view-fields at 200× magnification per animal was calculated for each group.

#### 4.1.3. Immunofluorescence Analysis

Immunofluorescence for vanin-1 was performed as reported previously [27]. Briefly, to suppress endogenous peroxidase activity and nonspecific binding, the deparaffinized sections were incubated with 3% hydrogen peroxide and protein-blocking solution for 5 min at room temperature, respectively. Then, these sections were incubated with the diluted anti-vanin-1 antibody (USCN Life Science Inc., Wuhan, China) (1:100 dilution) overnight at 4 °C, followed by reaction with components from a labeled streptavidin-biotin peroxidase kit (Dako, Carpinteria, CA, USA) that included 3-amino-9-ethylcarbazole color development. Sections were then lightly counterstained with hematoxylin. Images were acquired with a light microscope.

### 4.2. In Vitro Experiments

The human kidney cell line (HK-2) was obtained from the American Type Culture Collection (Manassas, VA, USA) and maintained in DMEM/F12 medium (Life Technologies, Carlsbad, CA, USA) supplemented with 10% FBS, 100 U/mL penicillin, and 100 μg/mL streptomycin. Cells were incubated at 37° in a humidified atmosphere containing 5% CO_2_. To stimulate renal I/R injury in vitro, HK-2 cells were cultured in a hypoxic environment with 1% oxygen (O_2_), 94% nitrogen (N_2_), and 5% CO_2_ in modular gas chambers for 24 h, followed by reoxygenation for 0, 1, 3, or 6 h in a 21% O_2_, 5% CO_2_, and 74% N_2_ incubator at 37 °C.

#### Cell Viability Assay

Cell viability was evaluated using the cell counting kit-8 (CCK-8) solution assay (Dojindo, Kumamoto, Japan). Briefly, HK-2 cells were seeded at a density of 1 × 10^4^ cells per well in 96-well plates. After various treatments, 10 μL of CCK-8 solution was added to the cells and incubated for 2 h following the manufacturer’s specifications. The cell viability was assessed by measuring the optical density at 450 nm using a microplate reader.

### 4.3. Laboratory Measurements

After urine and blood samples were centrifuged at 1000× *g* for 10 min, supernatant and serum samples were obtained. Urinary and serum creatinine concentrations were measured by the Jaffe method using a commercial kit (Wako Pure Chemical Industries, Osaka, Japan). Vanin-1 in the urine, serum, and renal tissues was measured using an enzyme-linked immunosorbent assay (ELISA) kit (USCN Life Science Inc.) The urinary supernatants were used for the measurement of NAG by the colorimetric method (Wako), KIM-1 using an ELISA kit (R&D Systems, Minneapolis, MN, USA), and NGAL using an ELISA kit (BioPorto Diagnostics, Gentofte, Denmark).

#### Quantitative Real-Time PCR

Total RNA was extracted from kidney tissue samples after homogenization or cell culture samples using RNeasy Mini Kit (QIAGEN, Valencia, CA, USA) according to the manufacturer’s instructions. Isolated total RNA was reverse-transcribed with PrimeScript RT Reagent Kit (Takara Bio Inc., Otsu, Japan). Real-time quantitative PCR was performed using each primer and the probe (TaqMan gene expression assays), with an Applied Biosystems StepOnePlus Real-Time PCR System (Life Technologies). To control for variation in the amount of cDNA available for PCR in the different samples, mRNA expression levels of the target sequences were normalized to the expression of an internal control, *glyceraldehyde-3-phosphate dehydrogenase* (*GAPDH*). The GenBank accession numbers, assay identification, and target exons were NM_053805.1, Rn00441533_g1, and 1-2 (rat *Snai1*); NM_017008.4, Rn99999916_s1, and 3-3 (rat *Gapdh*); NM_002133.2, Hs01110250_m1, and 3-4 (human *HMOX1*); and NM_002046.5, Hs99999905_m1, and 3-3 (human *GAPDH*), respectively. Data were analyzed using the comparative threshold cycle method.

### 4.4. Statistical Analysis

Data, presented as the means ± SE, were compared using one-way ANOVA followed by Dunnett’s test. A value of *p* < 0.05 was considered significant. All statistical analyses were conducted with GraphPad Prism, version 4.03 (GraphPad Software, Inc., San Diego, CA, USA).

## 5. Conclusions

For appropriate therapeutic intervention, detection of renal tubular damage at an early stage is clinically important. Our findings suggest that urinary vanin-1 might be a potentially sensitive biomarker of AKI induced by I/R in rodents. To further examine this, clinical studies involving AKI patients will be needed.

## Abbreviations

AKI	acute kidney injury
EMT	epithelial-mesenchymal transition
GPI	glycosylphosphatidylinositol
GSH	glutathione
γGCS	γ-glutamylcysteine synthetase
I/R	ischemia/reperfusion
KIM-1	kidney injury molecule-1
NAG	N-acetyl-β-d-glucosaminidase
NGAL	neutrophil gelatinase-associated lipocalin
ROS	reactive oxygen species
